# The Fatty Acid Compositions, Irritation Properties, and Potential Applications of *Teleogryllus mitratus* Oil in Nanoemulsion Development

**DOI:** 10.3390/nano14020184

**Published:** 2024-01-12

**Authors:** Wantida Chaiyana, Jirasit Inthorn, Suvimol Somwongin, Pimporn Anantaworasakul, Sawat Sopharadee, Pornnapat Yanpanya, Marina Konaka, Wasin Wongwilai, Pongsathorn Dhumtanom, Saranya Juntrapirom, Watchara Kanjanakawinkul

**Affiliations:** 1 Department of Pharmaceutical Sciences, Faculty of Pharmacy, Chiang Mai University, Chiang Mai 50200, Thailand; jirasit_i@cmu.ac.th (J.I.); suvimol_s@cmu.ac.th (S.S.); pimporn.a@cmu.ac.th (P.A.); sawat_so@cmu.ac.th (S.S.); 2 Center of Excellence in Pharmaceutical Nanotechnology, Faculty of Pharmacy, Chiang Mai University, Chiang Mai 50200, Thailand; 3 Multidisciplinary and Interdisciplinary School, Chiang Mai University, Chiang Mai 50200, Thailand; 4 Faculty of Pharmaceutical Sciences, Burapha University, Chon Buri 20131, Thailand; pornnapatypy@gmail.com (P.Y.); 61210036@go.buu.ac.th (M.K.); 5 Renewable Energy and Energy Efficiency Research Unit, Multidisciplinary Research Institute, Chiang Mai University, Chiang Mai 50200, Thailand; wasin.w@cmu.ac.th; 6 Herbs and Functional Products Research Unit, Multidisciplinary Research Institute, Chiang Mai University, Chiang Mai 50200, Thailand; pongsathorn.d@cmu.ac.th; 7 Chulabhorn Royal Pharmaceutical Manufacturing Facilities by Chulabhorn Royal Academy, Chon Buri 20180, Thailand; saranya.jun@cra.ac.th (S.J.); watchara.kan@cra.ac.th (W.K.)

**Keywords:** cricket, *Gryllus bimaculatus*, *Teleogryllus mitratus*, *Acheta domesticus*, nanoemulsion, required hydrophilic–lipophilic balance, fatty acid, Fourier transform infrared spectrometer

## Abstract

This study aimed to characterize and investigate the potential of the oils from *Gryllus bimaculatus*, *Teleogryllus mitratus*, and *Acheta domesticus* to be used in nanoemulsions. The oils were extracted by a cold press method and characterized for their fatty acid profiles. Their irritation effects on the chorioallantoic membrane (CAM) were evaluated, along with investigations of solubility and the required hydrophilic–lipophilic balance (RHLB). Various parameters impacting nanoemulsion generation using high-pressure homogenization were investigated. The findings revealed that *G. bimaculatus* yielded the highest oil content (24.58% *w*/*w*), followed by *T. mitratus* (20.96% *w*/*w*) and *A. domesticus* (15.46% *w*/*w*). Their major fatty acids were palmitic, oleic, and linoleic acids. All oils showed no irritation, suggesting safety for topical use. The RHLB values of each oil were around six–seven. However, they could be successfully developed into nanoemulsions using various surfactants. All cricket oils could be used for the nanoemulsion preparation, but *T. mitratus* yielded the smallest internal droplet size with acceptable PDI and zeta potential. Nanoemulsion was found to significantly enhance the antioxidant and anti-skin wrinkle of the *T. mitratus* oil. These findings pointed to the possible use of cricket oils in nanoemulsions, which could be used in various applications, including topical and cosmetic formulations.

## 1. Introduction

Edible insect species are currently considered good sources of protein, and along with notable levels of fat, fiber, and essential micronutrients, they have consequently been proposed to significantly contribute to global food security in the future [1]. Edible insects have been considered an attractive and nutritious alternative food source for larger vertebrate livestock, due to their high feed conversion efficiency and environmental sustainability [2]. Recently, edible crickets have emerged as notable insects, gaining attention for their potential contribution to humans and livestock [3]. Crickets have a high protein composition that ranges from 55% to 73% dry matter, as well as large fat levels that range from 4.30% to 33.44% dry matter, with polyunsaturated fatty acids (PUFA) accounting for around 58% of total fatty acid composition [3]. In addition, crickets contained a high mineral content and vitamins [3]. Various minerals, including calcium, potassium, magnesium, phosphorus, sodium, iron, zinc, manganese, and copper, as well as lots of vitamins, including vitamins A, B, C, D, E, and K, were also detected in the crickets [4]. Aside from being rich in various nutrients, insect farming offers numerous benefits, such as the ability to operate on a small scale, requiring minimal investment and lacking the need for advanced technology, being conducted in a limited space, boasting quick breeding cycles, and presenting low barriers to entry for individuals or businesses [5].

Crickets are members of the Gryllidae family (Insecta, Orthoptera), in which there are about 2400 recognized species [6]. However, three major species widely consumed and farmed in Thailand included *Gryllus bimaculatus* (two-spotted cricket or field cricket), *Teleogryllus mitratus* (ground cricket), and *Acheta domesticus* (house cricket)*. G. bimaculatus* is also the main cricket species used for research in the United States and Japan [6], whereas another worldwide popular species is *A. domesticus* [7]. In addition to being a good source of protein and essential amino acids, crickets are a good source of lipids and fatty acids, particularly PUFAs, which have advantageous health benefits, and were thus suggested to be incorporated into routine diets to maintain health [8]. Cricket oil possesses qualities that make it well-suited for both table oils and as an ingredient in food products [9]. As crickets offer an environmentally friendly avenue for producing high-quality bio-based materials, they could be an attractive source of fats and oils, which are extensively utilized as crucial components within skin care emulsions [10]. However, there has been limited research exploring the utilization of cricket oil in cosmetics or topical skincare products.

The oil derived from *A. domesticus* has been reported to contain significant amounts of Ω-3 and Ω-6 fatty acids, which, beyond their dietary and functional properties, have been recognized for their healthy skin-promoting effects, notably in reducing inflammatory processes and being potentially beneficial for treating atopic dermatitis, psoriasis, and acne [11,12]. Additionally, triglycerides, commonly found in natural oils, including those from crickets, typically act as emollients, softening the skin and indirectly moisturizing it by reducing transepidermal water loss [13]. Our previous study revealed that the oil extracted from *G. bimaculatus* exhibited promising cosmeceutical properties for the skin via antioxidant, anti-tyrosinase (anti-pigmentation), and anti-aging activities, without causing irritation or cytotoxicity [14]. However, it was limited to only one species of the crickets. Expanding the investigations to encompass diverse cricket species, particularly those commonly raised in farming (*G. bimaculatus*, *T. mitratus*, and *A. domesticus*), would yield comprehensive insights into the potential benefits of cricket oils in cosmetics. This broader approach would not only enhance our understanding of their applicability beyond food, but would also extend to cosmetic, personal care, and even pharmaceutical products. This comprehensive exploration could significantly broaden the use of cricket oils across multiple industries.

Additionally, our prior study revealed the successful development of nanoemulsions using *G. bimaculatus* oil to enhance its cosmeceutical potential [14]. However, a comprehensive exploration into the factors impacting nanoemulsion development was not conducted. Therefore, the aims of the present study were to extract the oils from various species of crickets as well as to investigate their chemical profiles, irritation potency, and pre-formulation properties. Nanoemulsions were also developed, and various factors affecting the generation of nanoemulsions, including different types and quantities of cricket oils and surfactants, as well as the addition of humectants in the formulations, were also investigated. The cosmetic efficacy of the cricket oils in nanoemulsions was assessed in comparison to native oils concerning their antioxidant and anti-skin wrinkle properties.

## 2. Materials and Methods

### 2.1. Cricket Materials and Cricket Oils Extraction

Three types of crickets, including *G. bimaculatus*, *T. mitratus*, and *A. domesticus*, were purchased in frozen form from a local farm in Chiang Mai, Thailand, during April 2023. The frozen crickets were dried using a vacuum freeze dryer (Kinetic Engineering Co., Ltd., Bangkok, Thailand) for 24 h until dryness. The dried crickets were then subjected to a cold pressing process to produce the oils using an oil extraction machine (FEA-100SS-M-H-TC, Energy Friend Ltd. Part., Chiang Mai, Thailand). Each of the cricket oils were kept in well-closed containers until further experiments.

### 2.2. Chemical Materials

L-Ascorbic acid, ferrous sulfate (FeSO_4_), oleanolic acid, 2,2-diphenyl-1-picrylhydrazyl (DPPH), 2,4,6-tris(2-pyridyl)-striazine (TPTZ), collagenase from *Clostridium histolyticum* (EC 3.4.24.3), synthetic peptide 2-furanacryloyl-Leu-Gly-Pro-Ala (FALGPA), N-tris(hydroxymethyl)methylglycine (tricine), acetic acid, hydrochloric acid (HCl), polyoxyethylene (20) sorbitan monolaurate (Tween^®^ 20), polyoxyethylene (20) sorbitan monooleate (Tween^®^ 80), polyoxyethylene sorbitan trioleate (Tween^®^ 85), soy lecithin, ethanol, and dimethyl sulfoxide (DMSO) were analytical grade and purchased from Sigma-Aldrich (St. Louise, MO, USA). Mineral oil, olive oil, glycerin, propylene glycol, butylene glycol, and sorbitol were cosmetic grade and purchased from Namsiang Co., Ltd. (Bangkok, Thailand).

### 2.3. The Determination of the Chemical Profiles of Cricket Oils

#### 2.3.1. Fatty Acid Methyl Ester-Gas Chromatographic/Flame Ionization Detector (FAME-GC/FID)

The fatty acid profiles of cricket oils were evaluated using an in-house method of Central Laboratory (Thailand) Co., Ltd. (Chiang Mai, Thailand), based on the Association of Official Agricultural Chemists (AOAC) official method 996.06, a standard procedure for the determination of total, saturated, and unsaturated fat [15]. Prior to analysis, the cricket oils were transesterified in methanol with boron trifluoride (BF_3_) to generate fatty acid methyl esters (FAMEs), which were then examined using capillary gas chromatography (GC) combined with a flame ionization detector (FID). The GC/FID analysis was performed using an Agilent Model 6890N, No. G1530N (Agilent Technologies, Inc., Santa Clara, CA, USA), equipped with a Supelco SP-2560 capillary column (Supelco, Inc., Bellefonte, PA, USA) with an injection port and a FID detector set at 250 °C. The initial temperature was set at 140 °C, held for 5 min, and increased with a rate of 3 °C/min to 250 °C, finally held for 17 min. The total run time was 55 min.

#### 2.3.2. Fourier Transform Infrared Spectroscopy (FT-IR)

The molecular composition of cricket oils was investigated using an FT-IR. The aliquot amount of each cricket oil was subjected to an alpha-II FT-IR spectrometer (Bruker, Karlsruhe, Germany), equipped with the single reflection diamond attenuated total reflection (ATR) module, and the IR spectrum was subsequently recorded across a range of wavenumbers, scanning from 400 to 4000 cm^−1^. The FTIR spectra were plotted, with transmittance represented on the Y-axis and the wavenumber (cm^−1^) plotted along the *X*-axis.

### 2.4. The Irritation Test of Cricket Oils Using the Hen’s Egg Chorio-Allantoic Membrane (HET-CAM) Test

The irritation properties of each cricket oil were investigated on the chorio-allantoic membrane (CAM) of hen’s eggs using the HET-CAM test, which would require no ethical approval when the hen embryo’s age was less than half of the total incubation period (21 days) [16]. In the present study, fertilized hen’s eggs, aged 7 to 10 days, were purchased from the Faculty of Agriculture, Chiang Mai University, Thailand. Each hen’s egg was incubated in an automated rotating egg incubator (manufactured by Nanchang Howard Technology Co., Ltd., Jiangxi, China) at a temperature of 37.5 ± 0.5 °C and relative humidity set at 62.5 ± 7.5%. A dental micromotor (Marathon-3 Champion, Saeyang Microtech, Daegu, Republic of Korea) coupled with a rotating cutting blade was used to create an opening in the eggshell above the air cell to facilitate the application of the test substance onto the CAM. Prior to the test, the CAM was moistened using a normal saline solution (NSS). Subsequently, 30 µL of the cricket oils were applied to the CAM and the irritation effects were immediately observed for 5 min. The first irritation signs, in terms of hemorrhage, lysis, and coagulation, on the blood vessels were assessed, and the onset of each irritation signs in s was recorded and used for the calculation of an irritation score (IS).

IS = ((301 − t(h) × 5)/300((301 − t(l)) × 7)/300((301 − t(c)) × 9)/300,(1)

where t(h) is the time of first vascular hemorrhage detected, t(l) is the time of first vascular lysis detected, and t(c) is the time of first vascular coagulation detected. Irritation classification was based on IS as 0.0–0.9, non-irritation; 1.0–4.9, slight irritation; 5.0–8.9, moderate irritation; and 9.0–21.0, severe irritation. The experiments were performed in duplicates.

### 2.5. Pre-Formulation Study

#### 2.5.1. The Solubility Test of Cricket Oils

Each of the cricket oils were evaluated for their solubility in various solvents, including ethanol, DMSO, mineral oil, and olive oil. In brief, an accurate amount of the oil was weighted and recorded. Subsequently, the solvents were gradually added dropwise until the oils were completely dissolved. A vortex mixer (Genie 2, Scientific Industries Inc., New York, NY, USA) was used for enhancing the solubility rate of the oils after the addition of each drop of the solvents. The amount of each solvent used for the oils to be completely dissolved was recorded. The solubility of each cricket oil was calculated using the following equation:

Solubility (mg/mL) = A/B,(2)

where A is the weight (mg) of the cricket oil and B is the volume (mL) of the solvent required for complete dissolution of the oils. The experiments were individually carried out, each replicated three times.

#### 2.5.2. The Determination of the Required Hydrophilic–Lipophilic Balance (RHLB) of Cricket Oils

The RHLB values of each of the cricket oils were evaluated using the method previously described by Chaiyana et al. (2017) [17]. Various combinations of polysorbate 80 (a hydrophilic surfactant, HLB = 15) and sorbitan oleate (a lipophilic surfactant, HLB = 4.3) were used to generate surfactant mixtures across the HLB range from 4.3 to 15.0. Each surfactant mixture, at a concentration of 5% *w*/*w*, served as the emulsifier in the emulsion formulation by blending with each cricket oils and water. The resulting mixtures were thoroughly blended using a vortex mixer (Genie 2, Scientific Industries Inc., New York, NY, USA) for 10 min. The external appearance, particularly the emulsion phase separation, was observed once after the preparation and after the formulations were kept at room temperature for 1, 12, and 24 h. The phase separation of each formulation after 24 h was recorded and reported as a percentage, calculated using the following equation:

Phase separation (%) = A/B,(3)

where A is the height of the excess water phase (bottom layer) and B is the height of the entire formulation. On the other hand, an emulsion on the top layer was analyzed for the internal droplet size using a zeta sizer (Zetasizerfi, Malvern Instruments Ltd., Malvern, UK). The experiments were individually carried out, each replicated three times. The RHLB values of each cricket oil were determined based on the formulation that exhibited the least phase separation and the smallest internal droplet size.

### 2.6. The Development of a Nanoemulsion Containing Cricket Oils

Various factors affecting the development of nanoemulsions were evaluated, including variations in cricket oil or surfactant types and concentrations, along with the addition of humectants in the formulation. In brief, a primary emulsion was firstly prepared by mixing the cricket oils with the water phase, comprising surfactants, with or without the humectant. The resulting mixture was then homogenized using a high-speed homogenizer (IKA^®^ T25 digital Ultra-Turrax, Staufen, Germany) set at 12,000 rpm for 5 min. The primary emulsion was then subjected to a high-pressure homogenizer (APV 1000, Wilmington, MA, USA) set at 300 bars for 7 cycles. To investigate the impact of various cricket oil types, the oils derived from *G. bimaculatus*, *T. mitratus*, or *A. domesticus* were used at the concentration of 5% *w*/*w*, while 5% *w*/*w* Tween^®^ 85 (Sigma-Aldrich, St. Louise, MO, USA) was used as an emulsifier. In addition, different concentrations of cricket oil, ranging from 1% to 5% *w*/*w*, were used in the development of the nanoemulsion to observe the influence of varying oil amounts. Various types of surfactants, including Tween^®^ 20 (Sigma-Aldrich, St. Louise, MO, USA), Tween^®^ 80 (Sigma-Aldrich, St. Louise, MO, USA), Tween^®^ 85 (Sigma-Aldrich, St. Louise, MO, USA), and soy lecithin, were used to observe the effect of varying surfactant types, while the influence of surfactant concentration was examined within the range of 1% to 5% *w*/*w*. The influence of humectants was also investigated after the addition of glycerin, propylene glycol, butylene glycol, and sorbitol to the formulations.

### 2.7. The Characterizations of Nanoemulsions Containing Cricket Oils

The nanoemulsions containing cricket oils were evaluated for their external appearance by visual inspection. In addition, the internal droplet size, polydispersity index (PDI), and zeta potential were analyzed using a zeta sizer (Zetasizerfi, Malvern Instruments Ltd., Malvern, UK). The findings were displayed as the average and standard deviation (SD) derived from a minimum of 10 measurements conducted on each sample. Additionally, the viscosity of the nanoemulsion containing cricket oils was evaluated using a rheometer equipped with an R/S spindle CC48 (Brookfield Engineering Laboratories, Inc., Middleboro, MA, USA) at 25 °C. 

### 2.8. The Stability Test of Nanoemulsion Containing Cricket Oils

The stability in terms of homogeneity of each nanoemulsion was evaluated by the centrifugation method [18]. The formulation was subjected to the microcentrifuge (MINI-10K+, Hangzhou Miu Instruments Co., Ltd., Hangzhou, China) set at 5000 rpm for 15 min. Subsequently, the external appearance, particularly in terms of phase separation, was observed by visual inspection.

### 2.9. The Determination of Biological Activities Related to Cosmetic Applications of Nanoemulsion Containing Cricket Oils 

#### 2.9.1. Antioxidant Activities

- Ferric Reducing Antioxidant Power (FRAP) Assay

The ferric reducing antioxidant power of nanoemulsion containing cricket oil was evaluated in comparison with the native cricket oil using an assay modified from Chaiyana et al. (2020) [19]. In brief, 20 µL of the nanoemulsion containing 5% *w*/*w* of cricket oil or 5% *w*/*w* of cricket oil in DMSO was mixed with 180 µL of FRAP solution, prepared by combining 0.3 M acetate buffer pH 3.6, 10 mM TPTZ in 40 mM HCl solution, and 20 mM ferric chloride solution. After the incubation in for 5 min, the UV absorbance was measured at 595 nm using a multimode microplate reader (BMG Labtech GmbH, Ortenberg, Germany). The results were reported in terms of FeSO_4_ and L-ascorbic acid equivalent concentration, which were calculated from the calibration curves of FeSO_4_ and L-ascorbic acid, respectively. The experiments were individually carried out, each replicated three times.

- 2,2-Diphenyl-1-Picrylhydrazyl (DPPH) Radical Scavenging Assay

The DPPH radicals scavenging activities of nanoemulsion containing cricket oil was evaluated in comparison with the native cricket oil using an assay modified from Chaiyana et al. (2020) [19]. In brief, 20 µL of the nanoemulsion containing 5% *w*/*w* of cricket oil or 5% *w*/*w* of cricket oil in DMSO was mixed with 180 µL of 167 mM DPPH aqueous solution. After the incubation in the dark for 30 min, the UV absorbance was measured at 520 nm by a multimode microplate reader (BMG Labtech GmbH, Ortenberg, Germany). The percentage of DPPH inhibition was calculated using the following equation:

DPPH inhibition (%) = [(A − B)/A] × 100,(4)

where A represented the absorbance of the mixture without nanoemulsion or cricket oil solution and B represented the absorbance of the mixture with nanoemulsion or cricket oil solution. L-Ascorbic acid was used as the positive control. The dose response curves were plot with the percentage of DPPH inhibition versus log concentrations of cricket oil both in nanoemulsion or solution. The IC_50_ was the calculated using GraphPad Prism version 8.0.1 for Windows (GraphPad Software, San Diego, CA, USA). The experiments were individually carried out, each replicated three times.

#### 2.9.2. Anti-Skin Ageing Activities

The anti-skin ageing activities of nanoemulsion containing cricket oil were evaluated in comparison with the native cricket oil by means of collagenase inhibition, using the method modified from Chaiyana et al. (2020) [19]. In brief, 20 µL of the nanoemulsion containing 5% *w*/*w* of cricket oil or 5% *w*/*w* of cricket oil in DMSO was mixed with 20 µL of 5 units/mL collagenase with an enzyme activity greater than 90% and 80 µL of Tricine buffer. After the incubation for 15 min, 40 µL of 2 mM FALGPA in tricine buffer pH 7.5 was added to start the reaction and the UV absorbance was immediately measured kinetically at 340 nm for 20 min by a multimode microplate reader (BMG Labtech GmbH, Ortenberg, Germany). The percentage of collagenase inhibition was calculated using the following equation:

Collagenase inhibition (%) = [(A − B)/A] × 100,(5)

where A represented the absorbance of the mixture without nanoemulsion or cricket oil solution and B represented the absorbance of the mixture with nanoemulsion or cricket oil solution. Oleanolic acid was used as the positive control. The dose response curves were plot with the percentage of collagenase inhibition versus log concentrations of cricket oil both in nanoemulsion or solution. The IC_50_ was the calculated using GraphPad Prism version 8.0.1 for Windows (GraphPad Software, San Diego, CA, USA). The experiments were individually carried out, each replicated three times.

### 2.10. Statistical Analysis

All data were presented as a mean ± SD. Statistical significance was evaluated through one-way analysis of variance (ANOVA), followed by Tukey’s post hoc tests, conducted using GraphPad Prism software version 2.01 (GraphPad Software Inc., La Jolla, CA, USA). In conditions where the comparison was between two separate sample groups, the data were analyzed by an unpaired sample *t*-test using GraphPad Prism software version 2.01 (GraphPad Software Inc., La Jolla, CA, USA). A *p* value < 0.05 was considered significant.

## 3. Results and Discussions

### 3.1. Cricket Materials and Cricket Oils

**Figure 1:**
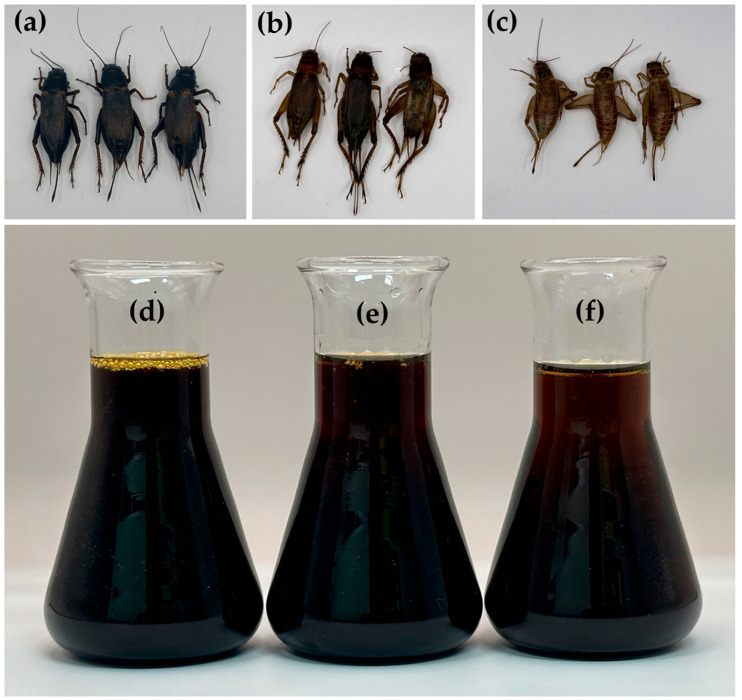
External appearance of *G. bimaculatus* (**a**), *T. mitratus* (**b**), *A. domesticus* (**c**), and the oils extracted from *G. bimaculatus* (**d**), *T. mitratus* (**e**), and *A. domesticus* (**f**).

Different types of crickets displayed distinct characteristics, as shown in Figure 1. The apparent differences among cricket species included variations in size and color. *G. bimaculatus* and *T. mitratus* have larger sizes compared to *A. domesticus*. Among the cricket species in the present study, *G. bimaculatus* exhibited the darkest color, followed by *T. mitratus* and *A. domesticus*, in descending order. The color of *G. bimaculatus* derives from a combination of melanin (brown/black) and sclerotin (yellow/tan) pigments within its exoskeleton cuticle [20,21,22]. A distinguishing feature of *G. bimaculatus* is the distinct yellow spotted pattern prominently displayed on its forewings, hence earning it the common name “two-spotted cricket” [20]. The Thai common name for *G. bimaculatus* is “Tong Dam”, where “Tong” signifies gold and “Dam” denotes black color, reflecting its coloration. On the other hand, *T. mitratus* displays a scarlet color with reddish or brownish tones [23], thereby acquiring the common Thai name “Tong Dang”, which translates to copper, depicting its resemblance to this hue. Aligned with the others, *A. domesticus* manifests a brown-black color [24], but in a lighter tonal variation compared to *G. bimaculatus* and *T. mitratus*.

As crickets are recognized for their high fat content [3,25], they were used as a source for the oil production in the current study. Oils were successfully extracted from *G. bimaculatus*, *T. mitratus*, and *A. domesticus*, as shown in Figure 1. All cricket oils were dark brown liquids, exhibiting slight variations in color based on the respective cricket species. The oil derived from *G. bimaculatus* was the darkest, followed by *T. mitratus*, and *A. domesticus*, respectively. *G. bimaculatus* was noted as a cricket that yielded the highest oil content, with a yield of 24.58% *w*/*w* of its dried matter, followed by *T. mitratus* (20.96% *w*/*w*) and *A. domesticus* (15.46% *w*/*w*), respectively. These findings were in line with a previous study by Udomsil et al. (2019), which reported that *G. bimaculatus* yielded a higher fat content than *A. domesticus*, with a lipid content of 23.4 ± 0.1% and 10.4 ± 0.1% dry matter, respectively [26]. In addition, the findings about the yield of *T. mitratus* oil were also related to a previous study by Grabowski et al. (2021), which reported a crude fat content range of 22.71% to 24.32% of dry matter for *T. mitratus* [27]. However, the fat yield of crickets could be varied depending on various factors, such as diet, rearing environment, and extraction methods [28,29,30]. The fat yield of *A. domesticus* has been observed to vary, ranging from 9.8% to 22.8% *w*/*w* of dry matter in some studies [30].

### 3.2. The Chemical Profiles of Cricket Oils

The chemical compositions of each cricket oil, as shown in Table 1, exhibit variations in the fatty acid profile among the samples. An abundance of unsaturated fatty acids (USFA) was detected, with an almost equal distribution between monounsaturated fatty acids (MUFA) and polyunsaturated fatty acids (PUFA). *T. mitratus* oil was found to contain the highest amount of MUFA (31.28 ± 0.01% *w*/*w*), followed by the oils derived from *G. bimaculatus* (29.65 ± 0.01% *w*/*w*) and *A. domesticus* (29.14 ± 0.01% *w*/*w*), respectively. On the other hand, *A. domesticus* was found to contain the highest amount of PUFA (33.59 ± 0.01% *w*/*w*), followed by the oils derived from *G. bimaculatus* (31.32 ± 0.01% *w*/*w*) and *T. mitratus* (30.99 ± 0.01% *w*/*w*), respectively. Additionally, the amount of saturated fatty acid (SFA) in each cricket oil was more abundant than that of MUFA and PUFA. The findings also highlighted the variations in specific fatty acids among the oils derived from different crickets. The notable prevalence of palmitic acid was observed in *G. bimaculatus* oil (29.82 ± 0.01%), cis-9-oleic acid in *T. mitratus* oil (30.56 ± 0.01%), and cis-9,12-linoleic acid in *A. domesticus* oil (32.45 ± 0.01%). However, these three fatty acids (palmitic acid, cis-9-oleic acid, and cis-9,12-linoleic acid) were noted to be the primary fatty acid components presented in all cricket oils. The findings aligned with previous studies regarding these three fatty acids as primary components. However, variations in the order of abundance among palmitic acid, oleic acid, and linoleic acid introduce specific differences, yet these three fatty acids consistently remain the primary constituents. For example, Chaiyana et al. (2023) reported that *G. bimaculatus* oil was rich in linoleic acid (31.08 ± 0.00%), followed by oleic acid (30.44 ± 0.01%) and palmitic acid (24.80 ± 0.01%) [14], whereas Gan et al. (2022) reported that the oil was rich in linoleic acid (41.75 ± 0.23%), followed by palmitic acid (24.31 ± 0.14%) and oleic acid (22.75 ± 0.09%) [31].

**Figure 2:**
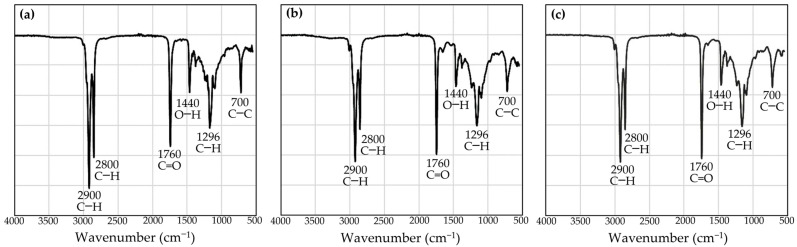
FR-IR spectra of the oils derived from *G. bimaculatus* (**a**), *T. mitratus* (**b**), and *A. domesticus* (**c**).

As the Fourier transform infrared spectrometer (FT-IR) offers advantages over conventional qualitative and quantitative analysis methods in terms of ease of sample handling, shorter analysis duration, reduced solvent use, improved signal-to-noise ratio, simultaneous recording of all wavelengths, and no need for high energy [32], it was used for chemical profile characterization of each cricket oil. The FT-IR spectra of each cricket oil are shown in Figure 2. FT-IR identifies oil composition via unique bands at characteristic frequencies corresponding to molecular vibrations of specific functional groups. All cricket oils were found to have the same fingerprint. The outstanding bands were found around the wavenumber of 2900, 2800, 1760, 1440, 1296, and 700 cm^−1^. The band around 2900 and 2800 cm^−1^ was assigned to the C−H stretching vibration of the methylene (−CH_2_) and methyl (−CH_3_) groups, which could be the alkyl groups of triglycerides found in the oils [33,34]. Previous studies revealed that absorbance peaks in the 3000−2800 cm^−1^ region have been detected in various oils, including petroleum and vegetable oils [35,36]. Another major band that was detected in the cricket oils at around 1760^−1^ corresponds to the stretching of the C=O bond in the carboxylic acid group. This peak has also been reported as a significant characteristic of the esters from lipid triglycerides and fatty acids [37]. On the other hand, the peak around 1440^−1^ was assigned to the in-plane O−H bending mode [38,39], whereas the peak around 1296 cm^−1^ was assigned to the C−H stretching [40]. Cricket oils also showed a band around the wavenumber of 700 cm^−1^, which could be assigned as C−C aromatic bending vibrations, which has been previously reported to be observed between 900 and 700 cm^−1^ [41,42]. While the fingerprint of each cricket oil appeared similar, the intensity of the band around 2900 and 2800 cm^−1^, corresponding to the C−H stretching vibrations of −CH_2_ and −CH_3_ groups, was notably more pronounced in the oil derived from *G. bimaculatus* compared to the others. This observation was consistent with its fatty acid profile, wherein SFA were found to be the most abundant in *G. bimaculatus* oil, particularly palmitic acid.

### 3.3. Irritation Properties of Cricket Oils

Natural oils have been widely used in cosmetic formulations as emollients due to their properties to soften the skin by creating an occlusive oil film on the stratum corneum, and preventing transepidermal water loss [43]. Moreover, natural oils could replace the main lipid components of the skin after the lipid bilayers were damaged by solvents, soaps, and harsh dry or cold weather conditions. Fatty acids were also known as one of the skin’s lipid components, along with ceramide and cholesterol. Additionally, various fatty acids, including palmitic, oleic, and linoleic acids, were used as base components of the oil phase of many cosmetic formulations [44]. Cricket oils contain beneficial fatty acids ideal for cosmetic formulations. Despite this, they are seldom utilized topically in cosmetics, raising concerns about their safety and potential for skin irritation. The current study investigated the irritation potency of each cricket oil using hen’s egg-chorioallantoic membrane (HET-CAM) tests. Although originally being intended as an alternative method for assessing eye irritancy [45], the HET-CAM test has also been equivalent to direct experiments on human skin. Wilson and Steck (2000) have confirmed consistent results for a particular substance (15% lactic acid) in both HET-CAM and direct skin tests [46].

**Figure 3:**
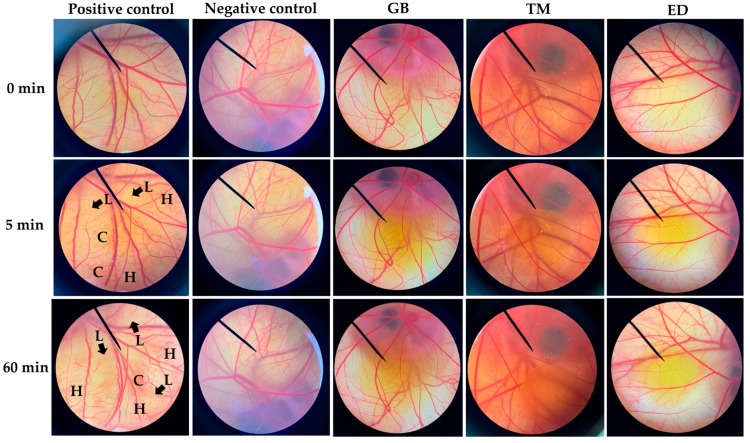
Photographs from the HET-CAM test illustrate the CAM before exposure to the test sample (0 min) and after exposure to a positive control (1% *w*/*v* sodium lauryl sulfate aqueous solution), negative control (normal saline solution), and oils extracted from *G. bimaculatus*, *T. mitratus*, and *A. domesticus* for 5 min and 60 min. The letter “H” represents vascular hemorrhage, “L” represents vascular lysis, and “C” represents vascular coagulation.

The CAM exposures to each cricket oil are shown in Figure 3, and their irritation scores are provided in Table 2. The findings highlighted that all cricket oils were safe, as they did not induce any irritation signs. In contrast, various irritation signs were observed on the CAM applied with the positive control, which was 1% *w*/*v* sodium lauryl sulfate aqueous solution. Hemorrhage observed as bleeding from blood vessels within the CAM, vascular lysis detected as the disappearance of small blood vessels, and coagulation observed as intravascular or extravascular clotting that resulted in the increased opacity of the CAM were observed within 5 min, and were more severe with the longer exposure time. The robust irritation response exhibited by the positive control, scoring 19.9 ± 0.0, in contrast to the absence of irritation from the negative control (normal saline solution), serves as validation for the findings derived from the HET-CAM test.

### 3.4. The Solubility of Cricket Oils

**Figure 4:**
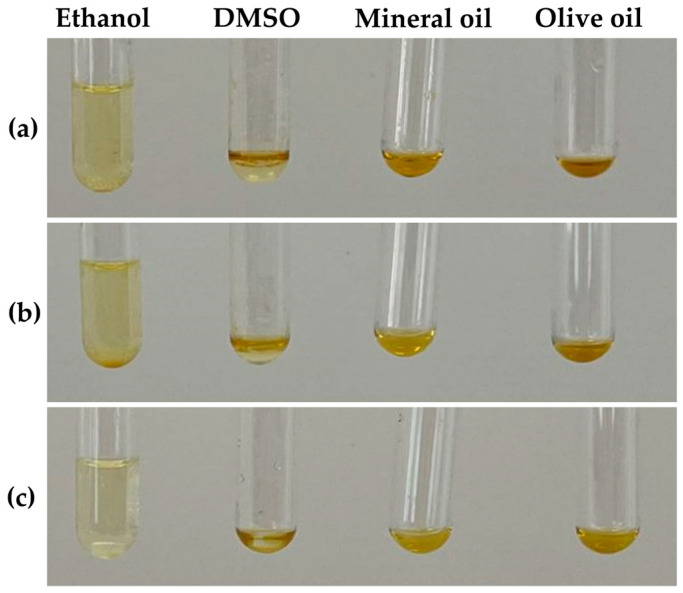
Photographs from the solubility test illustrate the lowest amount of ethanol, dimethyl sulfoxide (DMSO), mineral oil, and olive oil that completely solubilized the oils extracted from *G. bimaculatus* (**a**), *T. mitratus* (**b**), and *A. domesticus* (**c**).

Solubility is one of the important parameters not only to achieve the desired biological or pharmacological response [47,48], but also for formulation development. The solubility of cricket oils in various solvents are shown in Figure 4 and Table 3. All cricket oils were found to be freely soluble in DMSO (ε = 46.90 [49]), mineral oil (ε = 2.1 [50]), and olive oil (ε = 3.065 [51]). In contrast, they were found to be sparingly soluble in ethanol (ε = 25.04) [52]. However, variations were observed in the solubility of different cricket oils across various solvents. The oil derived from *A. domesticus* was found to be the most soluble oil among all cricket oils in DMSO, mineral oil, and olive oil.

### 3.5. RHLB Values of Cricket Oils

The alignment between the RHLB of oils and the HLB of surfactants in the formulation development is crucial for compatibility and effective emulsification. Achieving the correct HLB value ensures the proper emulsification, stability, and functionality of the formulation, all of which have a direct influence on its efficacy in delivering the medicine or active component [53]. The smallest droplet size of the emulsions derived from the formulation with the optimum HLB of the emulsifier blend [54]. Hence, determining the RHLB of cricket oils would be an important pre-formulation approach, particularly for the nanoemulsion development.

**Figure 5:**
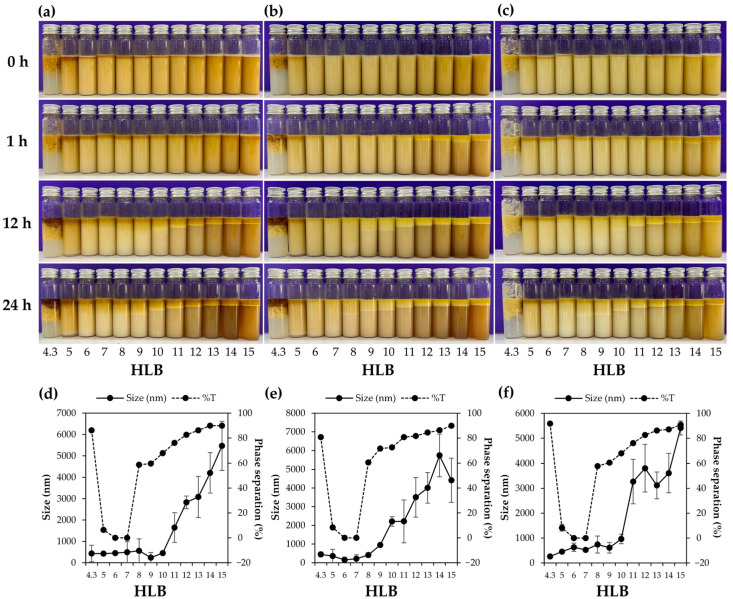
The external appearance of emulsions, composed of a mixed emulsifier with HLB values ranging from 4.3 to 15.0, water, and varying cricket oils, including *G. bimaculatus* (**a**), *T. mitratus* (**b**), and *A. domesticus* (**c**), observed at 0, 1, 12, and 24 h after preparation. Additionally, the size of internal droplets and the percentage of phase separation in each emulsion derived from *G. bimaculatus* (**d**), *T. mitratus* (**e**), and *A. domesticus* (**f**), were evaluated after 24 h.

The external appearance of emulsions emulsified using different HLB values of the surfactant blend is shown in Figure 5. Emulsions containing the oil derived from *T. mitratus* showed the highest degree of homogeneity compared to the other oils when observed immediately after preparation. Nevertheless, upon prolonged standing, phase separation became evident within each formulation. The formulations with the emulsifier with HLB values of six–seven were found to be homogeneous, even after prolonged standing for 24 h. Moreover, the internal droplet size of these formulations was found to be the smallest, indicating the alignment of the HLB value to the RHLB of the oils [54]. Therefore, it can be concluded that the RHLB values for all cricket oils were consistent, averaging between six and seven.

### 3.6. Nanoemulsion Containing Cricket Oils

**Figure 6:**
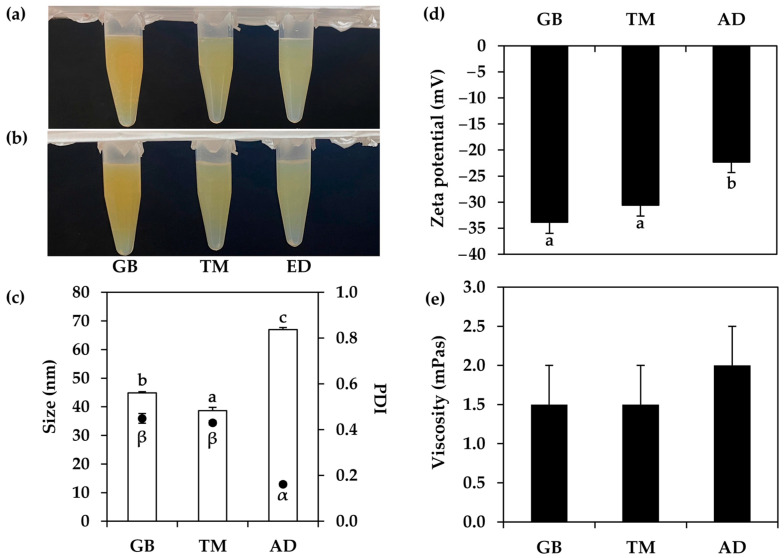
External appearance of nanoemulsions containing oils derived from *G. bimaculatus* (GB), *T. mitratus* (TM), and *A. domesticus* (AD) before (**a**) and after (**b**) centrifugation at 5000 rpm for 15 min, internal droplet size (☐) and polydispersity index (PDI, ●) (**c**), zeta potential (**d**), and viscosity (**e**) of each nanoemulsions. The letters a, b and c, as well as the symbols α and β, denote statistically significant differences among nanoemulsion formulations.

Various cricket oils were used for the development of nanoemulsions, as shown in Figure 6. All nanoemulsions were found to be translucent liquids with different colors depending on their oil phase. The viscosity of each nanoemulsion was found not to be significantly different. The oil derived from *G. bimaculatus*, which has the darkest color, also yielded the nanoemulsion with the most yellowish color. On the other hand, nanoemulsions from the oils derived from *T. mitratus* and *A. domesticus* had about the same appearance. Despite the initial dark color of the oils, the resulting nanoemulsions from cricket oils exhibited a pale to slight yellow color. Even in the case of the nanoemulsion derived from *G. bimaculatus* oil, known for its darkest color, the resultant nanoemulsion exhibited a satisfactory yellowish color. The likely explanation could be attributed to the use of oils at a low concentration of 5% *w*/*w*. Additionally, it could be due to the inherent light-scattering properties of oil droplets in nanoemulsions, often resulting in an opaque to translucent appearance that could potentially mask the dark color of the internal oil droplets [55].

Nanoemulsion stability assessment is critical in product development. Achieving long-term stability for cosmetic products, with a 3-year shelf life, proves to be a challenging and costly step in formulating new products [56]. Alternatively, utilizing centrifugation, which accelerates potential destabilization processes, offers a means to decrease the quantity of stored formulations and their storage duration [56]. In the current study, all nanoemulsions were found to be stable, as they remained homogeneous after centrifugation, which was used to induce product instability, simulating ageing and enabling a rapid evaluation of emulsion stability [57]. 

Aside from the external appearance of each nanoemulsion, the internal droplet size, PDI, and zeta potential were found to be different. The oil derived from *G. bimaculatus* yielded a nanoemulsion with the significantly smallest internal droplet size of 38.69 ± 1.11 nm (*p* < 0.05). The results aligned with previous findings, indicating that *G. bimaculatus* oil consistently produced the most homogeneous emulsion when combined with emulsifier systems with different HLB values within the emulsion system. Nevertheless, the nanoemulsion containing oil derived from *A. domesticus* exhibited the narrowest PDI, but had the least pronounced negative zeta potential value. PDI has been used to assess the quality of nano formulations in terms of particle size distribution, which describes the degree of non-uniformity in a particle size [58]. PDI values greater than 0.7 suggested an extremely broad particle size distribution, and were most likely ineligible for analysis using the dynamic light scattering (DLS) approach [58]. On the other hand, narrow PDI values suggested a uniformity of the particle size in the formulation, which would reflect the stability of the formulation. PDI values smaller than 0.4 indicated the homogeneity and uniformity of the formulations [59]. Hence, the nanoemulsion containing cricket oils, with PDI values ranging from 0.162 ± 0.009 to 0.449 ± 0.021, remained within an acceptable range.

Aside from the PDI value, the zeta potential was also the most studied parameter used for the prediction of formulation stability [60]. Zeta potential reflects nanodroplet charge, with higher positive or negative values signaling repulsion among droplets with similar charges. This phenomenon helps prevent coalescence and phase separation within the nanoemulsions. Hence, zeta potential significantly influences nanoemulsion stability, where those with a zeta potential exceeding ±30 mV were considered stable [61]. Although the nanoemulsion with *A. domesticus* exhibited the narrowest PDI, it displayed the least pronounced zeta potential (−22.30 ± 2.02 mV). In contrast, the nanoemulsions containing oils from *G. bimaculatus* and *T. mitratus* showed zeta potentials within an acceptable range (−33.87 ± 2.12 and −30.60 ± 2.08 mV, respectively). Therefore, *T. mitratus* oil, which yielded the nanoemulsion with the smallest internal droplet size, acceptable narrow PDI, and zeta potential, was selected for use in further experiments.

**Figure 7:**
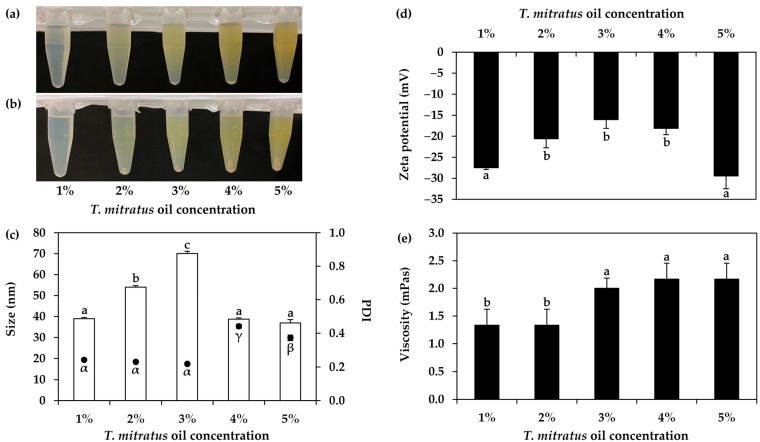
External appearance of nanoemulsions containing oils derived from *T. mitratus* at the concentrations ranging from 1 to 5% *w*/*w* before (**a**) and after (**b**) centrifugation at 5000 rpm for 15 min, internal droplet size (☐) and polydispersity index (PDI, ●) (**c**), zeta potential (**d**), and viscosity (**e**) of each nanoemulsions. The letters a, b, and c, as well as the symbols α, β, and γ, denote statistically significant differences among nanoemulsion formulations.

The impact of the concentration of an oil phase within the nanoemulsions were also evaluated, and the results are shown in Figure 7. All nanoemulsions were translucent but had different colors depending on the amount of oil phase. As *T. mitratus* oil had a dark brown color, the higher concentrations of *T. mitratus* oil resulted in nanoemulsions with a more yellowish color. All formulations were found to have the same physical appearance after centrifugation, reflecting the stability of the nanoemulsions. Aside from the external appearance, the concentration of an oil phase influenced the internal droplet size, PDI, zeta potential, and viscosity of the nanoemulsions. The viscosity was found to increase along with the higher concentration of the oils. On the other hand, distinct effects on the internal droplet size, PDI, and zeta potential were observed between the oil concentrations ranging from 1 to 3% *w*/*w* and those at 4% *w*/*w* and higher. An increase in oil concentration resulted in larger internal droplet sizes and less pronounced zeta potential up to a concentration of 3% *w*/*w*. However, beyond 4% *w*/*w*, the droplet size began to decrease, while the zeta potential became increasingly pronounced. PDI was found to remain narrow around 0.2, when the oil concentration was 3% *w*/*w* or lower, whereas the PDI showed an increase beyond 4% *w*/*w*. The findings about the internal droplet size corroborated a previous study on clove oil nanoemulsions, demonstrating that increased clove oil content (ranging from 2.5 to 15% *w*/*w*) led to a reduction in internal droplet size, decreasing from 145.3 ± 11.5 to 36.0 ± 6.2 nm [62]. Although the higher content of the oil phase led to a small internal droplet size, the viscosity of the whole formulation increased. Therefore, a suitable concentration of the oil phase needed to be optimized. The current study suggested a *T. mitratus* oil concentration of 5% *w*/*w*, as it generated the nanoemulsion with the significantly smallest internal droplet size, acceptable PDI, the significantly most pronounced zeta potential, and suitable viscosity for the high-pressure homogenizer (*p* < 0.05).

**Figure 8:**
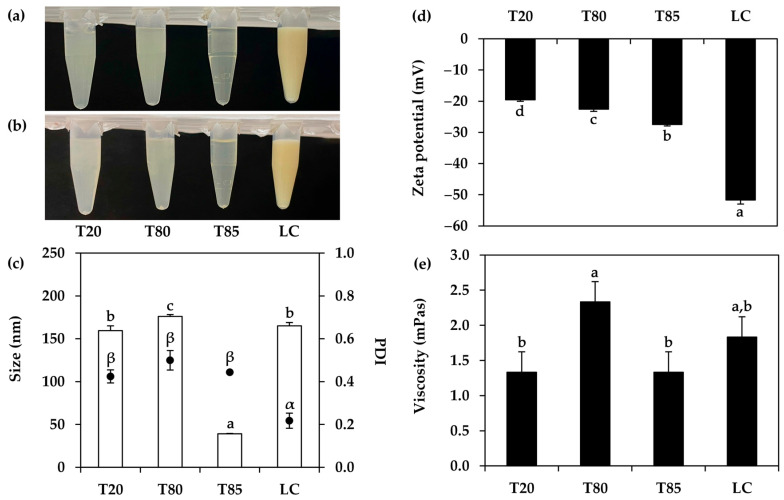
External appearance of nanoemulsions containing oils derived from *T. mitratus* and various surfactant types, including Tween^®^ 20 (T20), Tween^®^ 80 (T80), Tween^®^ 85 (T85), and lecithin (LC), before (**a**) and after (**b**) centrifugation at 5000 rpm for 15 min, internal droplet size (☐) and polydispersity index (PDI, ●) (**c**), zeta potential (**d**), and viscosity (**e**) of each nanoemulsion. The letters a, b, c, and d, as well as the symbols α and β, denote statistically significant differences among nanoemulsion formulations.

Apart from the influences of the oil phase, the effects of the emulsifier were also investigated. Nanoemulsions containing 5% *w*/*w* of *T. mitratus* oil with various surfactant types were successfully developed, as shown in Figure 8. Different surfactants, including Tween^®^ 20 (Sigma-Aldrich, St. Louise, MO, USA; HLB = 16.7 [63]), Tween^®^ 80 (Sigma-Aldrich, St. Louise, MO, USA; HLB = 15 [63]), Tween^®^ 85 (Sigma-Aldrich, St. Louise, MO, USA; HLB = 11 [63]), and lecithin (HLB = 3–5 [64]), were used as emulsifiers in the nanoemulsion systems. The nanoemulsion from Tween^®^ 85 (Sigma-Aldrich, St. Louise, MO, USA) was the most translucent, whereas that from lecithin was opaque and turbid. The external appearance of the nanoemulsions had a strong correlation with their internal droplet size, with Tween^®^ 85 (Sigma-Aldrich, St. Louise, MO, USA) emulsion having the lowest internal droplet size of 42.12 ± 1.05 nm, while the others varied from 174.90 ± 3.95 to 187.27 ± 6.07 nm. On the other hand, lecithin was found to yield the nanoemulsion with the most pronounced zeta potential value, due to the ionic structure of lecithin, which was due to the cationic charge of the trimethylammonium groups and the anionic charge of the phosphate groups [65]. Although phosphate and trimethylammonium groups neutralize each other, the cationic trimethylammonium was prominent at a low pH; thereby, the zeta potential, which was the ionicity surrounding the droplets, was negative. Among all surfactants used in the present study, Tween^®^ 85 (Sigma-Aldrich, St. Louise, MO, USA) was found to be most suitable, as it yielded the nanoemulsion with the significantly smallest internal droplet size, along with an acceptable PDI and zeta potential value. The likely explanation could be attributed to the relatively close HLB value of Tween^®^ 85 (Sigma-Aldrich, St. Louise, MO, USA), which was 11 [63], to the RHLB range of *T. mitratus* oil, which was 6–7. Despite lecithin having a closer HLB, which was three–five [64], to the RHLB of the *T. mitratus* oil, previous experiments emphasized that an emulsifier with an HLB of five or lower could not generate the emulsion.

**Figure 9:**
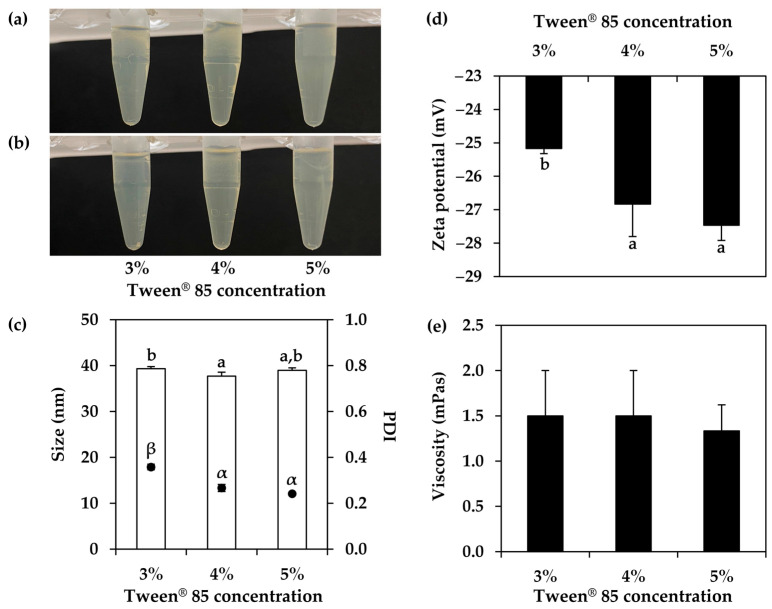
External appearance of nanoemulsions containing oils derived from *T. mitratus* and various concentrations of Tween^®^ 85, ranging from 3 to 5% *w*/*w*, before (**a**) and after (**b**) centrifugation at 5000 rpm for 15 min, internal droplet size (☐) and polydispersity index (PDI, ●) (**c**), zeta potential (**d**), and viscosity (**e**) of each nanoemulsion. The letters a and b, as well as the symbols α and β, denote statistically significant differences among nanoemulsion formulations.

The impacts of varying surfactant concentrations were evaluated, as illustrated in Figure 9. Nanoemulsions developed using Tween^®^ 85 concentrations ranging from 3 to 5% *w*/*w* exhibited uniformity in physical appearance, internal droplet size, and viscosity, showing no notable distinctions. All nanoemulsions were stable following centrifugation, indicating their stability. The tendency for PDI to be narrower and the zeta potential value to be more pronounced were detected with increasing surfactant concentrations. The results were in accordance with the previous study, which reported that the PDI of orange oil nanoemulsion had decreased from 0.97 to 0.6 with increasing Tween^®^ 80 ratio in the formulations [66]. However, the findings contrasted with some prior research that revealed a decrease in the internal droplet size of limonene nanoemulsions, from 99.50 ± 1.01 to 40.85 ± 0.58 nm, but had no influence on the PDI at greater Tween^®^ 80 concentrations ranging from 5 to 15% *w*/*w* [67]. Although different concentrations of Tween^®^ 85 had no effect on the internal droplet size and viscosity of the *T. mitratus* oil nanoemulsions, concentrations of 4% *w*/*w* and above were suggested, as they could produce nanoemulsions with a narrower PDI and a more pronounced zeta potential value.

**Figure 10:**
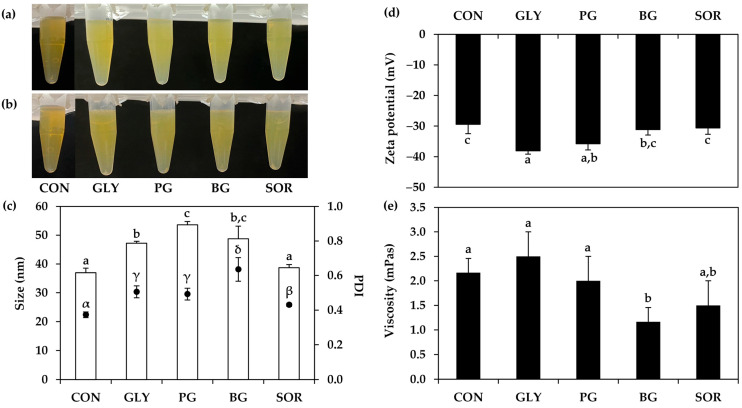
External appearance of nanoemulsions containing oils derived from *T. mitratus* without (CON) and with various types of humectants, including glycerin (GLY), propylene glycol (PG), butylene glycol (BG), and sorbitol (SOR), before (**a**) and after (**b**) centrifugation at 5000 rpm for 15 min, internal droplet size (☐) and polydispersity index (PDI, ●) (**c**), zeta potential (**d**), and viscosity (**e**) of each nanoemulsion. The letters a, b, and c, as well as the symbols α, β, γ, and δ, denote statistically significant differences among nanoemulsion formulations.

The impacts of glycol addition as a humectant in the nanoemulsion systems were also evaluated. The findings noted that the external appearance of nanoemulsions with and without glycol addition was similar, and all nanoemulsions were stable after the centrifugations, as shown in Figure 10. The internal droplet size was found to be larger after the addition of glycerin, propylene glycol, and butylene glycol, but not for sorbitol. Moreover, the PDI values were found to be broader, while the zeta potential values were slightly shifted to be more pronounced. The viscosity of each nanoemulsion was comparable, except in the case of butylene glycol, which had a lower viscosity. Our findings did not align with a prior study, which reported that adding polyethylene glycol (PEG) to the aqueous phase of nanoemulsions significantly reduced droplet diameter [68]. The likely explanation would be the variable effects of glycol on the viscosity of the nanoemulsions. Tabibiazar et al. (2015) previously discovered that PEGs increased aqueous phase viscosity, resulting in a considerable reduction in droplet diameter owing to improved droplet breakup efficiency [68]. However, the glycols used in the current investigation had no effect on viscosity and may have even lowered the viscosity. This variation in findings may serve to clarify the differences observed between the outcomes of the current study and those of prior research. The findings from the present study could be used to suggest sorbitol as the most compatible glycol used as a humectant in the *T. mitratus* oil nanoemulsion, since it had no effect on the external appearance, internal droplet size, zeta potential, and viscosity.

### 3.7. Biological Activities Related to the Cosmetic Applications of Nanoemulsions Containing Cricket Oils

**Figure 11:**
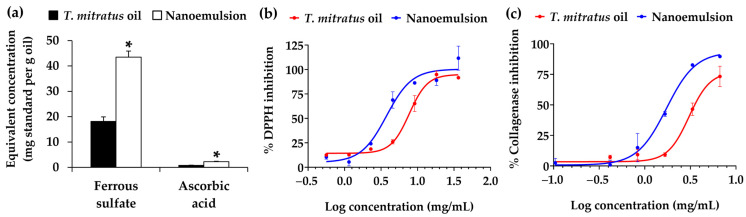
Ferric reducing antioxidant power (**a**) and dose response curves on the inhibition of DPPH radical (**b**) and collagenase activities (**c**) of *T. mitratus* oil and nanoemulsion containing 5% *w*/*w* *T. mitratus* oil. The asterisk symbol (*) denotes statistically significant differences between *T. mitratus* oil and nanoemulsion containing 5% *w*/*w* *T. mitratus* oil formulations (*p* < 0.05).

Biological activities relevant to cosmetic applications, focusing on the antioxidant and anti-skin aging properties of nanoemulsion containing *T. mitratus* oils, were investigated in comparison with its native *T. mitratus* oils, as shown in Figure 11. The antioxidant activities, involving the ferric reducing antioxidant power and DPPH radical scavenging properties, were both evaluated to confirm the antioxidant potential through distinct mechanisms. The FRAP assay was based on the capacity of antioxidants to transfer one electron in order to reduce metallic ions, whereas the DPPH assay was based on the ability of antioxidants to react with an organic radical through a transfer of both a hydrogen atom and an electron [69]. The nanoemulsion significantly increased the ferric reducing capacity of *T. mitratus* oils, displaying equivalent concentrations of 43.46 ± 2.40 mg FeSO_4_ per g of sample and 2.27 ± 0.14 mg L-ascorbic acid per g of sample. This is notably higher compared to the native *T. mitratus* oils, which exhibited equivalent concentrations of 18.12 ± 1.78 mg FeSO_4_ per g of sample and 0.78 ± 0.10 mg L-ascorbic acid per g of sample. The reducing power of the nanoemulsion was noted to be more than double that of the native *T. mitratus* oils. Similarly, the DPPH radical scavenging activity of *T. mitratus* oils was augmented by the nanoemulsion, exhibiting an IC_50_ value of 4.5 ± 1.7 mg/mL, approximately twice that of the potency of native *T. mitratus* oils (10.8 ± 1.5 mg/mL). 

Regarding the anti-skin wrinkle properties, nanoemulsions containing *T. mitratus* oils were evaluated for the collagenase inhibitory activities in comparison with its native *T. mitratus* oils. Alterations to collagen represent a significant contributor to the visible signs and clinical manifestations of aging skin [70]. Collagenase (matrix metalloproteinase-1: MMP-1) plays a crucial role in initiating the fragmentation of collagen fibrils at specific cleavage points, ultimately resulting in the degradation of the collagen fibril and skin wrinkles [70]. The current study revealed that *T. mitratus* oils inhibited collagenase with an IC_50_ value of 51.1 ± 12.8 mg/mL, while their potency was significantly enhanced to an IC_50_ value of 3.2 ± 0.7 mg/mL when incorporated into a nanoemulsion (*p* < 0.05).

## 4. Conclusions

Crickets have been successfully used as sources of natural oils, with yields ranging from 15.46 to 24.58% *w*/*w* among different cricket types. Palmitic, oleic, and linoleic acids were identified as the primary fatty acids presented in all cricket oils. The consistent chemical profile across all cricket oils were also verified by their identical FT-IR spectra. The oils were safe and suitable for use as ingredients in topical and cosmetic formulations, since they induced no irritation. Despite each oil having slightly different solubility, the RHLBs were all the same: around six–seven. The oil derived from *T. mitratus* was found to be the most suitable for nanoemulsion development, since it yielded the nanoemulsion with the smallest internal droplet size, an acceptable narrow PDI, and zeta potential. The antioxidant and anti-skin ageing properties of the oils were enhanced after incorporation into the nanoemulsions. The outcomes of this study propose the potential utilization of cricket oils within nanoemulsions, thereby expanding their application scope beyond food and feed to encompass a diverse range of applications, including topical and cosmetic formulations. Further exploration of the nanoemulsion via transmission electron microscopy (TEM) is recommended to visualize its internal structure and morphology. Additionally, the clinical evaluation to assess the anti-skin aging properties of the nanoemulsion containing *T. mitratus* was suggested.

## Figures and Tables

**Table 1:** Chemical compositions of the oils derived from *G. bimaculatus*, *T. mitratus*, and *A. domesticus*.

Fatty Acid Compositions		Amount (% *w*/*w*)		
		*G. bimaculatus*	*T. mitratus*	*A. domesticus*
Palmitic acid	(C16:0)	29.82 ± 0.01 ^a^	25.81 ± 0.01 ^c^	26.49 ± 0.01 ^b^
Heptadecanoic acid	(C17:0)	2.41 ± 0.00 ^a^	1.63 ± 0.00 ^b^	0.94 ± 0.00 ^c^
Stearic acid	(C18:0)	6.16 ± 0.00 ^c^	9.18 ± 0.00 ^a^	8.85 ± 0.00 ^b^
Arachidic acid	(C20:0)	0.52 ± 0.00 ^b^	0.67 ± 0.00 ^a^	0.50 ± 0.00 ^c^
Behenic acid	(C22:0)	0.08 ± 0.00 ^c^	0.43 ± 0.00 ^a^	0.12 ± 0.00 ^b^
Lignoceric acid	(C24:0)	0.03 ± 0.00 ^b^	0.00 ± 0.00 ^c^	0.09 ± 0.00 ^a^
Saturated fatty acids (SFA)		39.02 ± 0.01 ^a^	37.73 ± 0.01 ^b^	37.00 ± 0.01 ^c^
Palmitoleic acid	(C16:1n7)	0.80 ± 0.00 ^a^	0.36 ± 0.00 ^c^	0.39 ± 0.00 ^b^
Trans-9-Elaidic acid	(C18:1n9t)	0.21 ± 0.00 ^c^	0.33 ± 0.00 ^a^	0.30 ± 0.00 ^b^
cis-9-Oleic acid	(C18:1n9c)	28.60 ± 0.01 ^c^	30.56 ± 0.01 ^a^	28.64 ± 0.01 ^b^
cis-11-Eicosenoic acid	(C20:1n11)	0.05 ± 0.00 ^b^	0.04 ± 0.00 ^c^	0.08 ± 0.00 ^a^
Monounsaturated fatty acid (MUFA)		29.65 ± 0.01 ^b^	31.28 ± 0.01 ^a^	29.14 ± 0.01 ^c^
cis-9,12-Linoleic acid	(C18:2n6)	29.49 ± 0.01 ^b^	29.33 ± 0.01 ^c^	32.45 ± 0.01 ^a^
gamma-Linolenic acid	(C18:3n6)	0.65 ± 0.00 ^a^	0.14 ± 0.00 ^b^	0.00 ± 0.00 ^c^
alpha-Linolenic acid	(C18:3n3)	0.70 ± 0.00 ^b^	1.15 ± 0.00 ^a^	0.63 ± 0.00 ^c^
cis-11,14-Eicosadienoic acid	(C20:2)	0.03 ± 0.00 ^c^	0.13 ± 0.00 ^b^	0.25 ± 0.00 ^a^
cis-8,11,14-Eicosatrienoic acid	(C20:3n6)	0.12 ± 0.00 ^b^	0.14 ± 0.00 ^a^	0.04 ± 0.00 ^c^
Arachidonic acid	(C20:4n6)	0.06 ± 0.00 ^a^	0.00 ± 0.00 ^b^	0.00 ± 0.00 ^b^
cis-5,8,11,14,17-Eicosapentaenoic acid	(C20:5n3)	0.27 ± 0.00 ^a^	0.12 ± 0.00 ^c^	0.21 ± 0.00 ^b^
Polyunsaturated fatty acid (PUFA)		31.32 ± 0.01 ^b^	30.99 ± 0.01 ^c^	33.59 ± 0.01 ^a^
Unsaturated fatty acid (USFA)		60.98 ± 0.01 ^c^	62.27 ± 0.01 ^b^	63.00 ± 0.01 ^a^

NOTE: The letters a, b, and c, denote statistically significant differences among the cricket oils.

**Table 2:** Irritation score and irritation potency of the oils extracted from *G. bimaculatus*, *T. mitratus*, and *A. domesticus*.

Samples	Irritation Score	Irritation Potency
Positive control	19.9 ± 0.1 ^a^	Severe irritation
Negative control	0.0 ± 0.0 ^b^	No irritation
*G. bimaculatus* oil	0.0 ± 0.0 ^b^	No irritation
*T. mitratus* oil	0.0 ± 0.0 ^b^	No irritation
*A. domesticus* oil	0.0 ± 0.0 ^b^	No irritation

NOTE: Different letters, a and b, denote significant differences among the irritation scores of each sample, analyzed statistically using a one-way ANOVA and Tukey’s multiple comparisons test (*p* < 0.05).

**Table 3:** Solubility of the oils extracted from *G. bimaculatus*, *T. mitratus*, and *A. domesticus*.

Solvents	Solubility (mg/mL)		
	*G. bimaculatus* Oil	*T. mitratus* Oil	*A. domesticus* Oil
Ethanol	21 ± 2 ^c^	35 ± 1 ^a^	28 ± 1 ^b^
Dimethyl sulfoxide	144 ± 26 ^b^	161 ± 4 ^b^	551 ± 12 ^a^
Mineral oil	241 ± 21 ^b^	188 ± 3 ^c^	547 ± 22 ^a^
Olive oil	318 ± 7 ^c^	340 ± 10 ^b^	484 ± 7 ^a^

NOTE: Different letters, a, b and c, denote significant differences in the solubility among different cricket oils, analyzed statistically using a one-way ANOVA and Tukey’s multiple comparisons test (*p* < 0.05).

## Data Availability

Data available upon request.
